# Provings and Clinical Observations with High Potencies

**Published:** 1892-07

**Authors:** Malcolm Macfarlan

**Affiliations:** Philadelphia


### PROVINGS AND CLINICAL OBSERVATIONS WITH HIGH POTENCIES.

MALCOLM MACFARLAN, M. D., PHILADELPHIA.

### BAPTISIA-TINC.$^{6M}$.

Cured sore throat, enlarged tonsils; caused chilly and feverish sensations followed by sweat; somewhat later symptoms like intermittent fever; aches from his finger ends to his toes; very weak, sensation of fullness in stomach; eyes sensitive to light; checked disposition to sweat in hot weather.

Passes water often, with scalding sensation; bulk of urine is clear, with film on it of variegated colors after standing; deposit of uric acid is seen after few hours in the bottom of the vessel; the whole larynx externally is very sore; speech and swallowing painful; feels as if she must expectorate; eyeballs sensitive; could not bear much light; soreness in right ear, and running down the neck; *neck feels so tired that she cannot hold herself easy in any position.*

### BARYTA-CARB.$^{CM}$.

Sore throat; inflamed and enlarged tonsils; cured chronic inflammation of cervical glands mostly on the right side; had been very obstinate and resisted other treatment.

### BELLADONNA$^{101M}$.

Given in cases of chronic conjunctivitis causes a sensation of burning in the eye, and feeling in the cheek bones as if scalded; has often helped the severe pain in cases of hip-joint disease.

Relieved chronic enlargement of cervical glands of many years' duration. Woman, aged thirty, troubled since childhood; glands very large; had been treated off and on for years without benefit.

Produced rash-like scarlet-fever with severe tonsillitis, in several cases.

Swollen lips and cheeks; pain over the eyes; thirst; sensation as of a swelling or lump in throat; vomiting, soreness in region of liver; bowels loose; sick and fainty very often; can scarcely keep anything in the stomach. Feeling of great pressure at middle of sternum.

Produced symptoms of mania with disposition to violence in a case of advanced pulmonary consumption.

With difficulty *keeps any food in the stomach;* too weak to hold his head up long; passes water freely; exceedingly cross; ill-tempered; drowsy; but cannot get sound sleep; bowels constipated very much; stools dark and hard; tongue coated white; uneasy; restless; rouses up and screams; starts and screams as if frightened; boy, aged six; verified in cases of other children; sick and fainty; vomits nearly everything; becomes at times as white as a sheet.

Has cured vomiting often, particularly the vomiting of pregnancy.

Relieved enuresis in two weeks occurring in a boy, aged thirteen, who had been troubled since infancy.

Aching pain in all the teeth like dull toothache; mostly on left side; teeth feel sore to touch; great thirst; does not care much for food; wants to drink, which does not satisfy; burning sensation in pit of stomach; water nauseates him.

### Benzoic-acid$^{11M}$.

Given and curative because of excess of phosphates in the urine. A severe, aching pain in occipital region, or cerebellum, was cured in a few hours, which had confined the young man to bed for three weeks.

### Borax$^{3M}$.

Pain across his forehead for three days, with giddiness; then it passed to top of his head; giddy continually; soreness both sides of his neck; extending over the front of his chest; very severe in the middle of his sternum; cramps in the calves of leg.

### Bromine$^{CM}$.

*Headache* all week; hammering sensation in the temples and top of the head; aching in both knee-joints; scanty urine; turbid, light in color; sensation of swelling in the throat; feels badly in a general way. Caused very sore throat; tonsils very red.

### BRYONIA-ALB.$^{108M}$.

Severe pain in pit of stomach; general distress in epigastrium; bowels that were constipated more regular; nausea and general aching, with soreness in muscles.

### BUFO$^{45M}$.

Severe sore throat; tonsils painful; swallowing at meals caused much suffering. Frequently verified.

### BURSA-PASTORIS$^{9M}$.

Throat slightly sore; dryness of mouth; tonsillitis, tonsils swollen out even on a line with the angle of the jaw in one case; teeth sensitive when she shuts down on them; *gums spongy*; neuralgic feeling in teeth; slight ranula appeared. The girl had menstruated the week before, scantily, but it came on very profusely; she previously had amenorrhœa for some months.

Slight sore throat; hoarse in the morning; peculiar pain between the end of sternum and umbilicus, like pricking of needles.

Slight headache; pharynx sore; pain in stomach, felt generally as if he would be sick; nauseated; lips slightly sore; his finger joints hurt him greatly, as if rheumatic, and his urine burned in passing; pain in his left *shoulder*, so great that he thought his neck and shoulder would break. An abscess appeared on his finger on the tenth day; he then stopped the medicine for eighteen days; on resuming it in water, the upper portion of his throat became very sore, and the gums of upper and lower jaw on the inside felt as if full of blisters; crampish pain in stomach; toes hurt him; of course he did not know he was proving, and was made very sick.

Swelling of the throat and face on left side, mostly; *violent fever; throat* painful; oppressed breathing during the fever; slight deafness and pain in the left ear; free discharge of blood and mucus from the left nostril; general bruised feeling.

### CALENDULA-OFF.$^{45M}$.

Urine greatly diminished in quantity; left internal ear ached severely; piercing headache; eyes pained him because of the headache.

### CHELIDONIUM-MAJUS$^{5C}$.

On sixth day, vomited his breakfast, then took a chill, which confined him to bed for a day. After that took no more medicine.

### CROTON-TIGLIUM$^{45M}$.

Vomiting; very sore throat, felt mostly in the effort of swallowing; constant nausea and burning sensation in stomach.

### CICHORIUM-INTYBUS$^{5C}$.

Cured promptly two cases of hoarseness and pharyngitis; coughed a good deal night and morning.

### CALENDULA$^{45M}$.

Face puffy and swollen particularly under the eyes. Several provers.—Urine greatly diminished; left ear ached internally very much; discharge from ear; splitting headache; eyes pained him because of headache. Feels as if he would fall from a height when dropping asleep; urine diminished greatly in all the provers, male and female; less than half the usual quantity was passed.

### CANCER-FLUV.$^{5C}$.

It pains him to turn his eyes; giddy, nervous crawls all over his body. One prover, given every hour for a week.

### CAPSICUM-ANUUM$^{1M}$.

Produced a most severe attack of watery diarrhœa with cramps; neuralgic headache all over his head; coming on every little while; lasting but a short time; has to shut his eyes, aggravated by light.

### Carb-sulph.$^{45M}$.

Pain seizes her, and comes so quickly as to cause a sudden jerk; pain begins at left elbow and runs down the left side to the foot. One prover. Only permanent symptom developed after taking medicine a week.

### Caulophyllum-thal.$^{5M}$.

Produces hoarseness and loss of voice; caused pain and soreness in the ovarian region, mostly on left side; bearing-down sensation; frequently cures leucorrhœa, pain across sacrum, uterine and ovarian distress. In two cases of barrenness existing for years, when the medicine had been given for a long time, both conceived and bore children. I have found it a very reliable remedy in ovarian pain and soreness.

### Chamomilla$^{94M}$ (Anthemis).

One prover.—Child cries day and night; nothing pacifies it; disturbs everybody in the neighborhood; neighbors on both sides come in to see "what is up;" cries all night; had given it medicine every half-hour during the day for two days; frequently cured this symptom of crying in children with Chamomilla; child cries a great deal; cross and peevish without having any other ailment; no fever; fretfulness produced in a number of children.

### Chelon-glab. (F.)$^{45M}$.

Flesh was so sore it seemed as if the skin was off her elbow; other joints affected in a less degree. One prover.

### Calc-carb.$^{97M}$.

Frequently cured cases of marasmus in very young children with this remedy; they appear old, wasted, thin, especially those who had bronchitis without diarrhœa and without vomiting, when Sulph., Lyc., Laurocerasus, etc., did no good.

### Carbolic-acid$^{45M}$.

Promptly cured dry, scaly eczema of face, upper and lower extremities, of ten years' standing, which had resisted previous treatment of both schools.

### CADMIUM-SULPH.$^{5C}$.

Too frequent seminal emissions often checked by it. Not pushed as a proving.

### CHINA-OFF.$^{87M}$.

Exceedingly nervous and sensitive, pains generally all over frequently verified the symptom of over-sensitiveness to pain or noises.

### CICHORIUM-INTYBUS.$^{5C}$.

Neuralgia down the side of the neck, right side mostly.

### CIMICIFUGA-RAC.$^{95M}$.

Medicine given for three weeks every two hours caused pains all through her lower limbs, something of the character of growing pains in young persons, only a great deal worse; complete loss of appetite, *backache*, in small of back; fever every afternoon between twelve and four; menses (usually very regular) delayed three weeks, coughs at night, sleeps badly, *legs mostly affected*; heaviness in lower extremities.

### CLEMATIS-VIRG.$^{5C}$.

Have given this often with success in orchitis.

### COFFEA-CRUDA$^{70M}$.

Quickly cured a case of violent hysterical convulsions in a young girl. The disease had been of long standing. Patient had been confined to room and bed for some weeks. No other remedy given.

### COLLINSONIA-CAN.$^{45M}$.

Distress in the epigastrium; feeling of weight and pressure, with flatulency; disposition to piles.

### CHLORAL-HYDRAT.$^{CM}$.

Caused constant urination, attended with pain and scalding sensation; nervous, irritable, and restless.

### Conium-maculatum$^{3M}$.

Troublesome, teasing cough, no expectoration.

### Camphor$^{80M}$.

Promptly helped many times the watery diarrhœa of children, sudden nausea, with severe profuse vomiting ; inferior to Creosote in cholera infantum.

### Cornus-florida$^{45M}$.

Neuralgic sharp pains began on right elbow, extending to the hand and shoulder, passing down the right side and then up the left ; pain settled about the heart, causing feeling of pressure and palpitation ; couldn't use the arm because of pain and lameness, hands and feet appeared swollen, and the pains were of a darting, needle-like kind, very severe ; difficulty of passing water, no force to it. Left eye very weak, waters readily, vision appears slightly obscured ; elbows and wrist pain her, aching at the waist, as if she would break in two, to use her own language ; *sighs very often*, gagged as if she would vomit ; frequently in the morning chilly sensations come on, although she does not wish to be covered ; pains run up the whole left side of the trunk, or body, like lightning, seems as if it would give her a twist or jerk while coming on. *Sweat* just rolls down from her, chilly, warm sensation, alternating with cramps from the sides of her waist running toward the pubes, sleepy but could scarcely sleep, all night. Had to get up and look out of the window at night, she was so sleepless ; couldn't sleep in the day-time, continual slight perspiration.

### Cresotum$^{CM}$ (Beechwood).

Produces an isolated pimply rash on the face in many provers. I have made remarkable cures in both sexes of chronic periodical headaches in frontal region, piercing pain, where welts or swellings would come on the scalp after the suffering existed some time ; not useful in congestive headaches. Small red papules about the lower part of the face and neck ; remarkably curative in ailments of teething, particularly in hot weather; vomiting and frequent green stools.

### CUPRUM-ACET.$^{45M}$.

*Cramp in stomach*, but mostly low down toward the inguinal region, as if the parts would open, or, as if she would be torn apart. Slight headache in the temples, worse at junction of *occipital with both parietal bones;* aching pain in head, and scalp sore to touch; feels as if she had a load through the *apex of both lungs.*

Inner side of knees feel sore, and ankles hurts her, tongue coated with thick fur. Very disagreeable taste, bowels rather costive, which were loose; somewhat sleepless; aching and soreness down the *outside of the left thigh and leg* from the hip to the foot, right not much affected.

Sharp pains, with soreness to touch on both malar bones. *Throat very sore,* hurts her severely, tries to raise phlegm but gets up little, taste is like something putrid, singular feeling all over her, don't last long, can't explain it, feels as if she is moving without making any personal effort. Feels as if *her back at the top of her sacrum would break,* severe pain and cramp in abdomen. She is always worse in afternoon and evening and better in forenoon.

### CUPRUM-MET.$^{3M}$.

Male prover. *Backache* at top of sacrum, severe griping and spasm about the region of the heart, head troubles him with a confused feeling.

### CACTUS-GRAND.$^{CM}$.

Feeling about the heart as if it were compressed, anxiety, sighing, breathing, short breathing on the least exertion.

### COBALT-CHLORIDE$^{CM}$.

Tingling sensations in the feet as if asleep, sometimes like pricking of needles; circulation imperfect.

### CAUSTICUM$^{30M}$.

Profuse ropy, clear discharge from vagina, pain in her left hip-joint, commenced in an instant, as if her hip was suddenly hurt, soreness to touch on the front of the forearms, but lower extremities mostly affected; symptoms of a bad cold, coughed much, then a constant sniffling discharge from the nose, eyes water a good deal, light and heat of the fire hurt her eyes, feels sometimes as if there was excessive heat in her forehead. *Coughed so much* she thinks it affected her bowels, made them sore, cough strains her whole body.

Cured a large syphilitic (?) ulcer on scrotum, throat became sore, appetite diminished. Complained of constant coldness, and had shivering sensations while taking the remedy. Severe pain at each parietal protuberance, with repetition of general symptoms mentioned in paragraph above.

Given to a prover, relieved obstinate constipation, bowels now move more regularly and almost daily.

### CIMICIFUGA-RAC.$^{95M}$.

Relieved pains in abdomen, uterus, and distress about the heart, also severe bearing-down sensation, occurring in a pregnant woman who had been suffering a long time.

### CARB-VEG.$^{76M}$.

Caused watery diarrhœa, with griping on fourth day, and lasted three days; acute pain from right shoulder to elbow, extremity quite lame.

Slight urethritis in the male. Lacks sufficient confirmation to be sure of this.

### CINA$^{12C}$.

Violent coughing, lasting a great part of the night, as if she should strangle; lacking confirmation, but believed to be reliable because a similar cough has been often relieved by this remedy.

### CAMPHOR$^{80M}$.

Hemorrhage from the nose, occurring once a day or oftener; difficult to check, weakens him very much, never troubled so before; verified this often in many provers of Camphor. It is one of the most effective remedies in persistent nose-bleeding.

### CUPRI-ACETAS$^{45M}$.

Cured an old case of scrofulous ophthalmia in a child when other remedies failed. This I have often verified.

### COCHLEARIA-ARM.$^{10M}$.

Profuse painless diarrhœa, not sick with it, hungry feeling, unnatural craving for food.

### COPAIBA$^{CM}$.

Given in water every two hours produced after several days symptoms of strangury in a male; caused burning sensation in pharynx and tickling cough without expectoration.

### DIGITALIS-PURPUREA$^{CM}$.

Produced throbbing in every part of the body when touched; free diarrhœa; choking when he tries to swallow; thirst; great weakness in chest; constant disposition to void urine; does not wish to use his voice, because of feeling of exhaustion in chest.

### DIGITALIS$^{CM}$.

He coughs constantly at night, three hours at a time, as he states.

Many provers say they cough a great deal day and night; raise phlegm; never did cough so before.

### DRACONTIUM-FŒTIDUM$^{10M}$.

Cured severe aching pain in her *left* shoulder, constant belching and rumbling of wind in abdomen. The peculiar noise in the abdomen was like the gurgling of water, and could be heard over the room.

### DOLICHOS-PRURIENS$^{2C}$.

Head feels as if it would separate, it pains so, aching through the apices of both lungs, bruised pain in left inguinal region, soreness below the umbilicus.

The prover, a child with spinal curvature, had been subject to severe intestinal colic, occurring every few weeks, and which began five years ago; cure of colic was permanent and complete. The other symptoms developed were not recorded, because not confirmed by further provings.

### DORYPHORA-DECEMLINEATA$^{45M}$.

Soreness in and oppression of the chest; inflamed throat.

### EUPATORUM-PERFOLIATUM$^{CM}$.

Oppression at the middle of the sternum; feels as if something was pressing against his heart; palpitation; pains her to get and take a full breath; chest oppressed; symptoms of bronchitis; sharp pain in the eyes as if needles were being inserted; attacks of chills in the morning, no fever, unable to get warm, so chilly; eyes not inflamed; throat dry; becomes at times hoarse and loses her voice; oppression in chest very great; sharp burning pain in the feet; could not keep her shoes on sometimes while pain in feet lasted; feet seemed swollen; frequent short, dry cough; sharp pain in left ankle, hip, and shoulder, come on instantly and go away as quickly; weak and sick.

*Hoarseness.*—Prover, aged forty-five, general muscular soreness, worse in left ankle, left hip, left shoulder; *hoarseness* very great, can hardly talk; loses his voice; the trouble has passed down into his chest; hoarseness is greatest in the morning when he gets up; sudden violent contraction of the muscles of the right cheek; symptoms confirmed as to hoarseness and chest distress.

### ERIGERION-CAN.[45M].

Checked uterine hemorrhages of long duration when other remedies did no good. This I have verified many times. Turpentine high has about the same effect.

### ERECTHITES-HIERACIFOLIA[10M].

Stitches in the middle of the back; cold feeling in the back and legs.

Pain and soreness across the lumbar region.

Sore throat; legs feel stiff and painful, and aching across the small of her back.

### EUPION[45M].

Region of stomach felt internally sore and distended; not external soreness; pains under both short ribs.

### EUONYMUS-ATROP.[10M].

Urine increased; passes a great quantity at a time.

### ETHER-SULPHURIC[6C].

Remarkable effect in quickly curing severe neuralgia of the head in a woman aged sixty; been confined to bed weeks with it; had raised welts or swellings on scalp like ridges, accompanying the pain.

### EUPATORUM-PERF.[CM].

Sharp pain through the right chest when he breathes deeply; feels at night as if he was going out of his mind; disturbed breathing frightens him; effect of the medicine after a week.

### FERRUM-MET.[80M].

Believed to control hemorrhages in one disposed to consumption; had slight bleedings every week or two, but ceased entirely on exhibition of the remedy.

Nausea; vomited daily for three days after taking the medicine, never did so before; stopped the proving.

Rash like measles in a woman aged thirty-seven; hands felt as if she couldn't shut them because of a swollen sensation.

Controlled or stopped slight hemorrhages from the lungs and daily spitting of blood in young girl.

### FERRI-CARB.$^{50M}$.

Child aged seven years, four days' proving. Face suddenly red and purple, then pale; rest of body cold; extremities affected in a similar manner, alternating with slight fever and chilliness; changes are very rapid; stopped the medicine; have verified this in other provers.

### FILIX-MAS.$^{1M}$.

Lost his appetite; if he eats anything he is inclined to reject it soon after being swallowed; giddiness.

### FORMICA-RUFA$^{45M}$.

Pains down the anterior surface of both arms; worse about the elbow, as if bruised; felt as if she was going to smother; throat very sore; eyes and head very much affected; difficult to think or use her eyes well; chest internally felt badly from the nipples upwards; as if she would raise a quantity of mucus; sensation as if she would have quinsy; left side of throat worse than right; cough very troublesome; cough strains her; it is so severe and constant.

Lower extremities felt as if she had no power in them; sore and tired sensation across the abdomen below the navel, as if bruised; tired feeling in the back; felt slightly giddy.

### FLUORIC-ACID$^{45M}$.

Pain in eyeballs; hacking cough; sharp pain through temples.

### Ginseng$^{46M}$.

Soreness across his abdomen ; severe pain on either side of the top of the head, which caused him to shut his eyes ; ringing in his ears ; hiccoughing all day, off and on.

Sneezing ; blowing his nose ; became hoarse coughing; sensation of cold in chest; sharp pain in lungs, bowels loose, griping pain in abdomen.

### Guaiacum$^{5C}$.

Violent fever; her face became spotted, red blotches; eyes, nose, and cheeks have swollen appearance; tight, dry cough. muscles and joints of extremities sensitive.

### Glanderine$^{12C}$.

Feet feel sore and tired ; pains her every step she takes, particularly on the soles of her feet ; right arm very sensitive ; anterior surface of the elbow sore to touch ; no nose or throat symptoms developed that were prominent.

### Gummi-gutti.

Left ear ringing constantly ; sometimes hissing sensation, as of blowing off steam ; right ear slightly affected.

### Gelsemium-semper.$^{CM}$.

Her thighs were sore and sensitive to the touch; left more sensitive than the right; pains were all relieved when in a perspiration.

This remedy cured the prover in three weeks. She had wry neck and was almost paralyzed ; worse on left side ; head drawn to the left side ; inability to move the thighs but little ; unable to get her hands to her head; had been in this way for several months ; the subject of regular treatment for nearly a year previous to taking Gelsemium. Symptoms of another prover—eyes water, and inflamed ; coughs a good deal during the night; left ear discharged a little; had not previously done so; caused very loose bowels ; prover a weak, nervous woman, with glaucoma.

### GETTYSBURG SPRING WATER$^{45M}$.

Bowels that were regular, now costive.

*Another*, bowels now loose, three to four times a day, that were costive; hoarse, dry cough, as if from a scraped condition of throat. This is the language of the prover.

Pains in both shoulders and left knee; seminal emissions.

Urine profuse.

Sleepy and drowsy during the day.

A good deal of pain in upper left chest in front; pains her when she takes a deep breath.

Verified over and over again its good effect in muddy urine excess of urates, in frequent urination, in hip-joint disease, and rheumatic gout.

### GRAPHITES$^{CM}$.

Curative in provers with tinea tarsi.

Improved dimness of sight, caused by unhealthy secretion about eyelids.

### HAMAMELIS-VIRG.$^{10M}$.

Knocking, hammering sensation over left eye; throbbing sensation within the head; flushed face; inability to think well.

### HYPERICUM-PERFOL.$^{45M}$.

Child nauseated; complains of great pain in stomach; sick whenever it eats; bowels loose, two to three times daily. Cured a prover having articular rheumatism (knees mostly) with great effusion around the joint, and muddy urine, which, in a few hours, looked like the settlings of beer.

Caused severe pain in the last phalanges of the fingers, mostly thumb, fore, and little finger. Constant eructations night as well as day. Verified frequently its curative action in articular rheumatism and pains, affecting small joints. Sores inside the nose, itchy, continually picking it. This symptom was noticed in many provers. Cured eructations, caused severe cholera morbus on the third day, which continued several days. Stye on lower left eyelid, muscles sore, bruised feeling. When she took her shoes off found her feet *much swollen*, urine greatly diminished in amount, remained so for three days after stopping medicine. *Fearful sharp pain in knees, could hardly touch them* (symptoms after two weeks, medicine eight times a day). This was the most prominent symptom. Next in importance was his head trouble—dull pain; pains shoot through his fingers as if they were becoming sore.

Another prover, rheumatic woman, never passed so much water in her life, had to get up five or six times in night; great quantities at a time. Gave her relief from rheumatism.

### Hellebore$^{CM}$.

Pain within the chest under the left nipple, general muscular soreness, forced her menses on before the time, had to get up at night to pass urine.

Another prover—cured painful straining, constant desire to pass water with burning; don't need to get up at night to pass it as formerly, cured that symptom.

Scanty urine in one prover, where the secretion has been free.

### Hepar-sulph.$^{CM}$.

Did no good given alone in a case of real croup, with some sore throat Spongia$^{2C}$ afterward quickly cured the case. Verified this action of Spongia often; partial loss of voice existed in these cases.

### Hydrophobinum$^{1M}$.

Fever, no chill, pulse continuously rapid, no appetite, thirst, very sleepy, constant desire for cold drinks, which are swallowed without difficulty.

### Hyoscyamus$^{CM}$.

Cured permanently a child which would sob and cry a great deal at night in her sleep without awaking; chronic symptom, slight ague like chill every other day at eleven o'clock, with fever and sweat. In several provers caused *stupefying headache* continuously, cannot bear the least noise or to be talked to, eyes sensitive to light.

IODINE$^{17M}$.

Nose discharges clear mucus; considerable fever, no appetite; hot, dry skin all day long, creepy sensation, face fiery-red, pain in occiput and temples, teeth sticky with adhesive saliva. Promptly curative in the marasmus of a child thirteen months old, who had with it a moist eczema about the anus and inside of thighs, and apthæ. Creosote was given later in case there was a disposition to diarrhœa.

